# Forward single‐cell sequencing into clinical application: Understanding of cancer microenvironment at single‐cell solution

**DOI:** 10.1002/ctm2.782

**Published:** 2022-04-26

**Authors:** Xuanqi Liu, Charles A Powell, Xiangdong Wang

**Affiliations:** ^1^ Department of Pulmonary and Critical Care Medicine Zhongshan Hospital, Fudan University Shanghai Medical College, Shanghai Institute of Clinical Bioinformatics; Shanghai Engineering Research for AI Technology for Cardiopulmonary Diseases Shanghai China; ^2^ Division of Pulmonary, Critical Care and Sleep Medicine Icahn School of Medicine at Mount Sinai New York City New York USA

## Abstract

Single‐cell RNA sequencing (scRNA‐seq) is considered an important approach to understand the molecular mechanisms of cancer microenvironmental functions and has the potential for clinical and translational discovery and development. The recent concerns on the impact of scRNA‐seq for clinical practice are whether scRNA can be applied as a routine measurement of clinical biochemistry to assist in clinical decision‐making for diagnosis and therapy. Pushing single‐cell sequencing into clinical application is one of the important missions for clinical and translational medicine (CTM), although there still are a large number of challenges to be overcome. The present Editorial as one of serials aims at overviewing the history of scRNA‐seq publications in CTM, sharing the understanding and consideration of the cancer microenvironment at the single‐cell solution and emphasising the objective of translating scRNA‐seq into clinical application. The dynamic characteristics and patterns of single‐cell identity, regulatory networks, and intercellular communication play decisive roles in the properties of the microenvironment, malignancy and migrative capacity of cancer cells, and defensive capacity of immune cells. The microenvironmental single‐cell transcriptomic profiles and cell clusters defined by scRNA‐seq have great value for exploring the molecular mechanisms of diseases and predicting cell sensitivities to therapy and patient prognosis.

There is a fast‐growing understanding of single‐cell transcriptomic, epigenomic proteomic, metabolomic and transomic phenomes due to the booming development of single‐cell biotechnologies. Clinicians have raised issues regarding the value of single‐cell biology, such as whether single‐cell measures and phenomes have a clear link and whether correlations with patient phenomes reflect disease specificity and severity, can monitor responses to therapy, and can be used for the identification and development of disease‐specific biomarkers.^1^, ^2^, ^3^

To address these issues, it has been proposed to establish an artificial intelligence single‐cell model with the learning capacity to integrate biological function and imaging. For single‐cell biology, single‐cell RNA sequencing (scRNA‐seq) is an important approach to explore transcriptomic profiles, signal regulation, heterogeneity, evolution, and cell–cell communication in single‐cell solutions. The goal is to dynamically reveal the expression, signal, interaction, location, and new functions of single molecules within a single cell in multidimensional, multilayer, multicrossing, and stereoscopic aspects. scRNA‐seq combined with spatial transcriptomics can demonstrate the positioning of transcriptomic alterations at single cells in human tissues and the association of different gene expression with pathological phenomes, and can reflect in situ cell–cell communication.^4^, ^5^ Such advances are considered critical for developing spatiotemporal molecular medicine.^6^, ^7^

The recent concerns on the impact of scRNA‐seq for clinical practice are whether scRNA can be applied as a routine measurement of clinical biochemistry for understanding disease properties and molecular mechanisms, assisting in clinical decision‐making for diagnosis and therapy, and predicting the sensitivity in response to target drugs and the prognosis of patients.^8^ This is one of the important missions for clinical and translational medicine (CTM), although the implementation of scRNA‐seq in routine clinical practice still has a long way to go and the large challenges to overcome. The present mini‐review aims to reflect on the history of scRNA‐seq publications in CTM, sharing our understanding and consideration of the selection of those articles, and emphasising our objective to translate scRNA‐seq into clinical application.

The importance of scRNA‐seq for clinical and translational medicine was recognised 8 years after the first publication of single‐cell whole‐transcriptome analysis that introduced a new field to be explored for understanding the heterogeneity among individual cells.^9^, ^10^ The initial concerns on scRNA‐seq for clinical research were how to optimise and apply single‐cell isolation, amplify the genome/transcriptome, apply next‐generation sequencing for human tissues (Figure 1A), characterise inherent properties of a single cell from the large scale of the human genome, transcriptome, or epigenome, and design clinical studies to define single‐cell‐based molecular mechanisms in clinical oncology, immunology, microbiology, neurobiology, and pathology. It is unclear whether scRNA‐seq has potential for future clinical applications. Recent research has now shown that outcomes from scRNA‐seq improve the translational knowledge of the brain with complex networks and specialised cell–cell communication and the definitive heterogeneity among brain cells, and has uncovered clusters of cell subpopulations within various brain regions.^11^ Single‐cell transcriptomic profiles provide new insights into monitoring gene signatures, cell markers, and intercellular functions to promote the discovery of function‐, cell‐, network‐, and disease‐specific biomarkers and to elucidate their specific functions and state (Figure 1). scRNA‐seq creates a new opportunity to translate the complexity of cell–cell communication into the identification of biology‐specific diagnostic markers and therapeutic targets, although there are still challenges for translating bioinformatic tools, data analyses, and study designs from development to disease.

**FIGURE 1:**
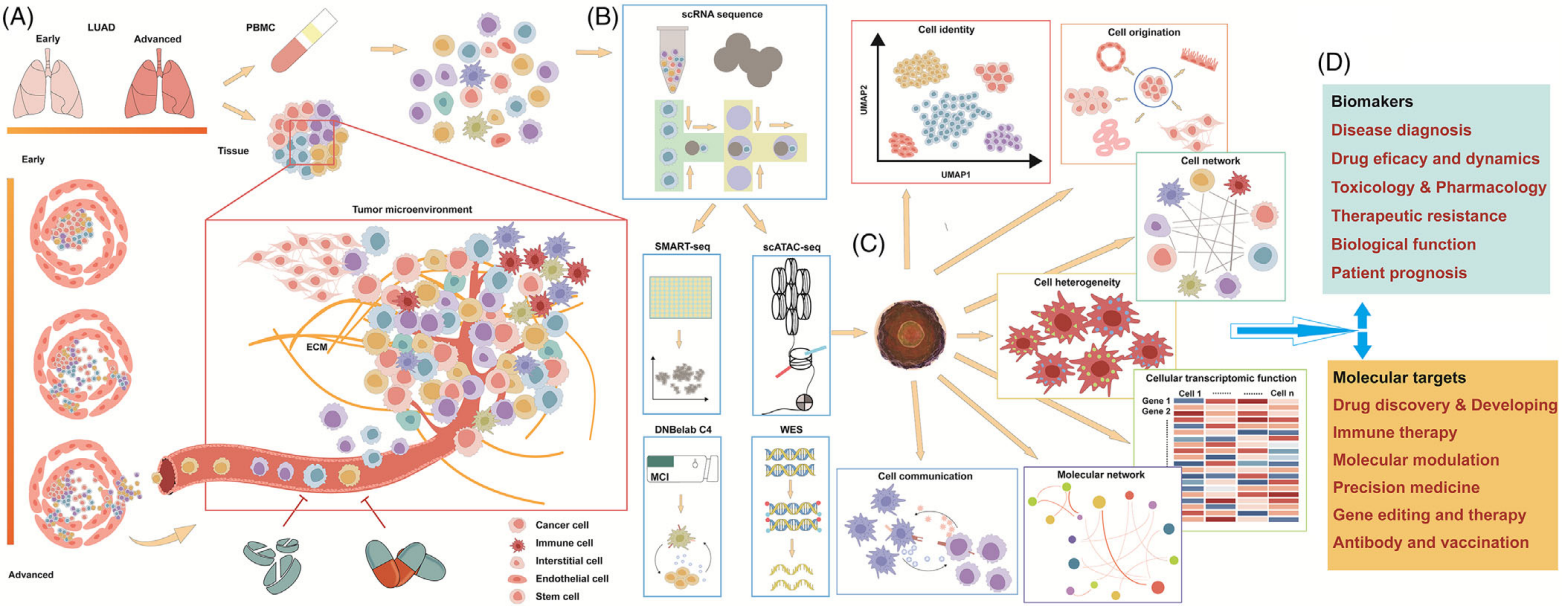
The workflow of the single‐cell atlas for understanding the tumour microenvironment. (A) Single cells were separated and isolated from samples of tumour tissue and peripheral blood for dynamic detection of the tumour microenvironment from the early to advanced stages of the disease, including the infiltration, invasion, and migration of tumour cells and immune cells. (B) Transcriptomic profiles, mutation, heterogeneity, and three‐dimensional (3D) genome are measured by RNA and DNA sequencing technologies combined with others, for example, from Smart‐seq to DNBelab C4 single‐cell RNA sequencing (scRNA‐seq), WES, and scATAC‐seq. (C) The detailed atlas of the tumour microenvironment was outlined at single‐cell resolution, for example, cellular identity, origination, network, heterogeneity, communication, molecular network, and cellular transcriptomic function. (D) The clinical application of scRNA‐seq is aimed at the determination of diagnostic biomarkers and therapeutic targets. ATAC‐seq, assay for transposase accessible chromatin with high‐throughput sequencing; ECM, extracellular matrix; LUAD, lung adenocarcinoma; PBMC, peripheral blood mononuclear cells; Smart‐seq, switching mechanism at the 5′ end of the RNA transcript; WES, whole exon sequencing

The processes of carcinogenesis, progression, and metastasis are critical steps of cancer development. scRNA‐seq together with single‐cell DNA sequencing demonstrate intratumor and intercellular heterogeneity, temporal and spatial heterogeneity, clonal evolution in primary tumors, cell invasion clusters in early‐stage cancers, trajectory of metastatic dissemination, and evolution of resistance to therapy.^12^ Data from scRNA‐seq comprehensively illustrate transcriptomic profiles, from which regulatory networks of differentiation, stemness, pluripotency, epithelial‐mesenchymal transition, and proliferation are defined. New clusters and molecular properties of stem cell‐like cancer cells have been uncovered to reveal molecular mechanisms by which cancer cells develop resistance during therapy, migrate from the origin to distant locations, and develop new mutations and epigenetic modification during evolution.

Recent studies have informed us of the advantages and disadvantages of methodologies for single‐cell isolation (Figure 1B), whole‐transcriptome amplification, and bioinformatics analysis of clinical samples (Figure 1C). For example, single‐cell analysis platforms offer a full image of heterogeneity in multidimensional functions and multiomics by integrating gene or protein expression, epigenetics, sequencing, phosphorylation, transcription, pathways, or interactions.^14^ Single‐cell transomics provides a dynamic, multilevel, multidimensional picture of molecular mechanisms in single cells to elucidate the regulation of gene–gene, gene–protein, and protein–protein interactions.^15^ Further work has advanced the translation of single‐cell multiomics into the field of clinical medicine,^13^ with a focus on regulatory networks and biological functions at multiple layers, directions, and angles of a single cell.

The microenvironment of cancer cells has important and critical roles in the maintenance of intercellular communication, nutritional support and extracellular haemostasis for cancer cells, immune cells, interstitial cells, and endothelial cells. The microenvironment elements, for example, cells, liquids, chemicals, and proteins, play decisive roles in the control of cancer cell differentiation, growth, migration, and response to therapy.^16^ scRNA‐seq provides comprehensive insights for understanding cell–cell communication, signalling, and regulation, for uncovering cell identity, and for defining the formation of the ecosystem that modulates tumour development. Wang et al. applied Smart‐seq2 and DNBelab C4 scRNA‐seq approaches to investigate the complex ecosystem of the cancer microenvironment with colorectal cancer as the primary cancer and hepatic metastases as the secondary cancer and to measure the proportion, location, cluster, and function of immune and nonimmune cells.^17^ This particular study demonstrated that B cells acted as pre‐B‐like cells expressing tumour suppressors at the early stage of primary cancer and developed into plasma cells at the advanced stage, which was clearly associated with the poor prognosis of patients with colorectal cancer. The analysis of cell–cell communication indicates that the high proliferation and interaction between IGLC2^+^ plasma cells and cycling B cells may have antitumoural effects in the control of cancer cell progression by recruiting CCR5^+^ T cells via CCL8 in primary tumors.

Multidimensional interactions between cancer cells and immune cells dominate the stereological mechanisms of the cancer microenvironment. Mei et al. integrated scRNA‐seq with whole‐exome sequencing and transposase‐accessible chromatin assays using sequencing (scATAC‐seq) to illustrate molecular communications between cancer cells, between immune cells, and between cancer cells and immune cells.^18^ The study focused on cancer cell tumour mutational burdens (TMB) with various transcriptomic profiles, epigenetic landscapes, and interactions with immune cells, although the mutation and transcriptomics were not measured in the exact same single cell. Within the microenvironment where 2/7 had high TMB and 5/7 had low TMB, the number of Th1/Th17 cells with CXCL13 and the expression of lymphotactin 1 and 2 (XCL1 and XCL2) bound to chemokine XC receptor 1 and 2 (XCR1 and XCR2) in CD8^+^ CTL and TEX cells were increased in high TMB.^18^ Patients with high TMB were more sensitive to the immune‐checkpoint blockade of PD‐1. However, it is still difficult to define transcriptomic profiles of single cells with high TMB because it is difficult to distinguish normal epithelia and cancer cells from the same microenvironment, to measure whole‐exome sequencing and scRNA‐seq simultaneously, and to uncover the direct association between mutation and regulatory network functions. Gao et al. distinguished normal cell types in the tumour microenvironment from malignant cells by measuring the copy number karyotyping of aneuploid tumours using an integrative Bayesian segmentation approach, with an accuracy rate of 98%.^19^ This is an important tool to translate scRNA‐seq into a diagnostic understanding of molecular functions and interactions between single normal and cancer cells. This makes it possible to detect TMB and transcriptomic profiles in the same single cell and to deeply investigate intercellular communication between normal cells, between cancer cells, and between normal cells and cancer cells.

Single‐cell transcriptomic profiles can be important and comprehensive molecular phenomes to differentiate various stages of disease and monitor cell sensitivity in response to therapy (Figure 1D). Chen et al. defined the early and advanced stages of lung adenocarcinoma according to single‐cell identity, network, and molecular features in the tumour microenvironment.^20^ The cancer microenvironment in advanced malignant lung adenocarcinoma was more complex and diverse, for example, more cancer‐associated fibroblasts, CD8^+^/CTL T cells, regulatory T cells, and follicular B cells, greater diversity of cell–cell communication genes, and other overexpressed genes associated with anti‐inflammation, innate immune response (e.g., TLR3, MYD88, and DDX5), as well as cell proliferation and differentiation (Figure 1A). The microenvironment dynamically changed with progression and showed upregulation of pathways for haemostasis, cell metabolism and metabolism, reactive oxygen species, inflammation, and angiogenesis, especially in epithelial cells. Zhou et al. investigated the influence of neoadjuvant chemotherapy in immune cell clusters and transcriptomics of the circulation and breast cancer tissue at a single‐cell solution.^21^ This particular study compared immune single‐cell transcriptomes and cell–cell communication between the circulation and cancer microenvironment, between circulating immune cells before and after therapy, and between microenvironmental immune cells of patients with different molecular subtypes (e.g., luminal A and B, HER‐2, or TNBC).

Comparative design is a common first step to compile single‐cell atlases of cancer tissues, for example, between the primary tumour tissue, adjacent noncancerous tissue (to the brim of matched tumour 3‐5 cm), and distinct normal tissues (to the brim of matched tumour ≥10 cm), between stages of disease, and between intracancer locations.^17^ Clinical phenome‐based validations of selected target genes/proteins mainly include the expression in human samples (e.g., tissue, blood, liquid), correction with severity, stage, and survival, sensitivity of therapeutic monitors in response to drugs, and specificity of the disease. Such validation can be performed in clinical samples or in public databases such as The Cancer Genome Atlas. One of great challenges is to select validation cohort patients who share similar characteristics of clinical phonemes, meet the requests of statistical analyses with enough numbers, and ensure the quality and reliability of validation data. In this particular study, scRNA‐seq was performed in 12 treatment‐naïve patients with colorectal cancer at stages I–IV, whole‐exome sequencing in seven patients, and scATAC‐seq in six patients.^18^ Clinical studies on immune single‐cell profiles can also provide valuable information for understanding the effects of immune cells within systemic and local environments on disease severity, stage, and response to therapy. For example, 18 patients with breast cancer were enrolled and divided into four groups based on luminal A and B, HER‐2, and TNBC subtypes, into two groups on biopsies and surgical sections, on different sites of the tumour, or pre‐ and postoperation, as well as into three groups on pre, mid‐, and posttreatments with drugs.^21^

In conclusion, scRNA‐seq is considered an important approach to advance the understanding of the molecular mechanisms of cancer microenvironmental functions and has the potential for clinical and translational discovery and development. The dynamic characteristics and patterns of single‐cell identity, regulatory networks, and intercellular communication play decisive roles in the properties of the tumor microenvironment, proliferation and migrative capacity of cancer cells, and antitumour capacity of immune cells. The microenvironment single‐cell transcriptomic profiles and cell clusters defined by scRNA‐seq have great value for exploring the molecular mechanisms of diseases, predicting cell sensitivities to therapy and predicting patient prognosis.
